# Classification of Ancient Mammal Individuals Using Dental Pulp MALDI-TOF MS Peptide Profiling

**DOI:** 10.1371/journal.pone.0017319

**Published:** 2011-02-25

**Authors:** Thi-Nguyen-Ny Tran, Gérard Aboudharam, Armelle Gardeisen, Bernard Davoust, Jean-Pierre Bocquet-Appel, Christophe Flaudrops, Maya Belghazi, Didier Raoult, Michel Drancourt

**Affiliations:** 1 Unité de recherche sur les maladies infectieuses et tropicales émergentes (URMITE), UMR CNRS 6236 IRD 198, IFR48, Faculté de médecine, Université de la Méditerranée, Marseille, France; 2 UMR 5140-CNRS, Université Montpellier III, Montpellier, France; 3 Direction régionale du service de santé des armées de Toulon, Toulon, France; 4 Ecole Pratique des Hautes Etudes and CNRS UPR 2147, Paris, France; 5 Centre d'Analyses Protéomiques de Marseille (CAPM), IFR Jean Roche, Faculté de Médecine - Secteur Nord, Université de la Méditerranée, Marseille, France; Natural History Museum of Denmark, Denmark

## Abstract

**Background:**

The classification of ancient animal corpses at the species level remains a challenging task for forensic scientists and anthropologists. Severe damage and mixed, tiny pieces originating from several skeletons may render morphological classification virtually impossible. Standard approaches are based on sequencing mitochondrial and nuclear targets.

**Methodology/Principal Findings:**

We present a method that can accurately classify mammalian species using dental pulp and mass spectrometry peptide profiling. Our work was organized into three successive steps. First, after extracting proteins from the dental pulp collected from 37 modern individuals representing 13 mammalian species, trypsin-digested peptides were used for matrix-assisted laser desorption/ionization time-of-flight mass spectrometry analysis. The resulting peptide profiles accurately classified every individual at the species level in agreement with parallel cytochrome *b* gene sequencing gold standard. Second, using a 279–modern spectrum database, we blindly classified 33 of 37 teeth collected in 37 modern individuals (89.1%). Third, we classified 10 of 18 teeth (56%) collected in 15 ancient individuals representing five mammal species including human, from five burial sites dating back 8,500 years. Further comparison with an upgraded database comprising ancient specimen profiles yielded 100% classification in ancient teeth. Peptide sequencing yield 4 and 16 different non-keratin proteins including collagen (alpha-1 type I and alpha-2 type I) in human ancient and modern dental pulp, respectively.

**Conclusions/Significance:**

Mass spectrometry peptide profiling of the dental pulp is a new approach that can be added to the arsenal of species classification tools for forensics and anthropology as a complementary method to DNA sequencing. The dental pulp is a new source for collagen and other proteins for the species classification of modern and ancient mammal individuals.

## Introduction

Classifying the remains of animals at the species level is an important task in various fields of research, such as zooarcheology, anthropology and related applied sciences, including forensic sciences and the surveillance of wildlife trade and endangered species [1]–[3]. This could, however be a challenging task, as species classification based on morphological characteristics lacks specificity and is of little use with fragmented or severely damaged material, such as a mixture of small skeletal pieces from several individuals [2]. Sequencing of specific genes and other metagenomic approaches using next-generation DNA sequencing technology are available and allow the classification of animal species. However, these approaches depend on the presence of amplifiable nucleic acids and time-consuming PCR-based methods that are prone to in-laboratory contamination [4].

Recent work has demonstrated that protein profiles obtained after matrix-assisted laser desorption/ionization time-of-flight mass spectrometry (MALDI-TOF MS) of cultured cells can accurately determine the species origin of the cell line [5], [6]. This approach is very promising, as MALDI-TOF MS is an easy-to-run and rapid method of analysis. Also, classification of animals has been done by MALDI-TOF MS of bone collagen [7]. Based on these seminal demonstrations, we hypothesized that MALDI-TOF MS could further classify mammal species by analyzing dental pulp tissue, i.e., a mixture of cell lines, instead of a unique cell line or protein.

To demonstrate that it was possible to classify animal remains, including ancient remains, at the species level starting from the dental pulp by using MALDI-TOF MS technology, we carried out a study into three successive sections combining MALDI-TOF MS expertise [5] with the experimental recovery expertise of dental pulp tissue [8]. First, we established that dental pulp was a source of peptide profiles identifiable by MALDI-TOF MS and MALDI-TOF MS dental pulp peptide profiling could be used for the accurate classification at modern mammalian individuals the species level. Second, we validated this method by a blind classification of modern mammalian teeth. Finally, we showed that MALDI-TOF MS analysis of dental pulp recovered from ancient buried mammalian individuals, including humans, enabled the rapid classification of these ancient individuals at the species level.

## Results

### Modern mammal database

We recovered 0.024–2.148 g/L of protein (mean ± standard deviation: 0.619±0.195 g/L) from 37 dental pulp tissues of 37 modern individuals representing 13 mammalian species. Because of the sole morphological examination of teeth could be misleading, species classification was confirmed in parallel by partial sequencing of the mitochondrial cytochrome *b* gene used as the gold standard (Table S1) [9]. We then digested extracted proteins by trypsin and used the resulting peptide mixture as the substrate for MALDI-TOF MS analysis. Same peaks that were observed in all of ten negative controls were contaminants composed of autoproteolytic trypsin digest fragments (1,726–2,284 Da) and keratin peaks (1,266–2,824 Da) [10], [11] and some additional non-specific peaks (707.3–3,807 Da) with a signal-to-noise ratio (SNR)≤3 were probably derived from the column used for the reverse-phase protein purification or from sample preparation. These peaks that we called contamination peaks were automatically eliminated from spectra obtained from dental pulp of modern mammals for generate a local modern mammal database. Spectra were derived from two to four dental pulp specimens per individual (one dental pulp per tooth). The peptide spectra had mass-to-charge ratio (m/z) of 700–4,000 Da and reproducibly yielded 11 to 42 semi-specific peaks (SEMPs) in the 1,000–3,000 Da (Table S2) that were present in all mammalian species studied. SEMPs and unique species-specific peaks (USSPs) were determined as previously published [10]. In silico analysis indicated that peptide spectra derived from the human (*Homo sapiens*) dental pulp contained six different collagen types. As for spectrum derived from the cow (*Bos taurus*), it contained 2 different collagen types. As for spectrum derived from the dog (*Canis familiaris*), it contained only collagen alpha-1(I) trypsin digestion products (Figure S1, Table S3). In the case of human dental pulp, peptide sequencing yielded 16 different non-keratin proteins including collagen (alpha-1 type I and alpha-2 type I) (Table S4). Applying the procedure (Materials and Methods), we then set up a 279-spectrum MALDI-TOF MS database of modern mammal dental pulp tissues to generate a modern mammal database. Spectra representing for each species have been deposited to Proteome Commons database https://proteomecommons.org/ [12] (Link: https://proteomecommons.org/tranche/data-downloader.jsp?h=9FYJgZ%2Bvzxy7TQtjfkLTPclKFDMB4uRYbbs9OgimjQaVCfAy2%2FXhPKzS42hWSVoyxhTRVRMBJsbdx26afXq1trhjC7cAAAAAAAAIgw%3D%3D) (Supplementary data online).

### Blind classification of modern mammal individuals

Protein samples from 37 modern individuals were coded, were digested by trypsin and were applied to the procedure MALDI-TOF MS. A total of 370 reproducible MALDI-TOF MS dental pulp profiles (10 per dental pulp) obtained in this time were directly compared to the local modern mammal database by using the software MALDI Biotyper 2.0 (Materials and Methods). Positive classification was not achieved in 3/37 (8.1%) dental pulp specimens with a protein concentration<0.1 mg/mL in the case of *Cavia porcellus* (2 teeth) and *Rattus rattus* (1 tooth), whereas 33/37 (89.1%) dental pulp specimens with a protein concentration>0.1 mg/mL (*P*<0.05, χ^2^ test) were accurately classified. In one *Vulpes vulpes* specimen, classification was of 1.436 below our classification threshold for modern mammal (2.0); in another *Vulpes vulpes* specimen, lack of classification was due to a low quality of the MALDI-TOF MS spectra (Table S5).

### Classification of ancient mammal individuals

**Figure 1:**
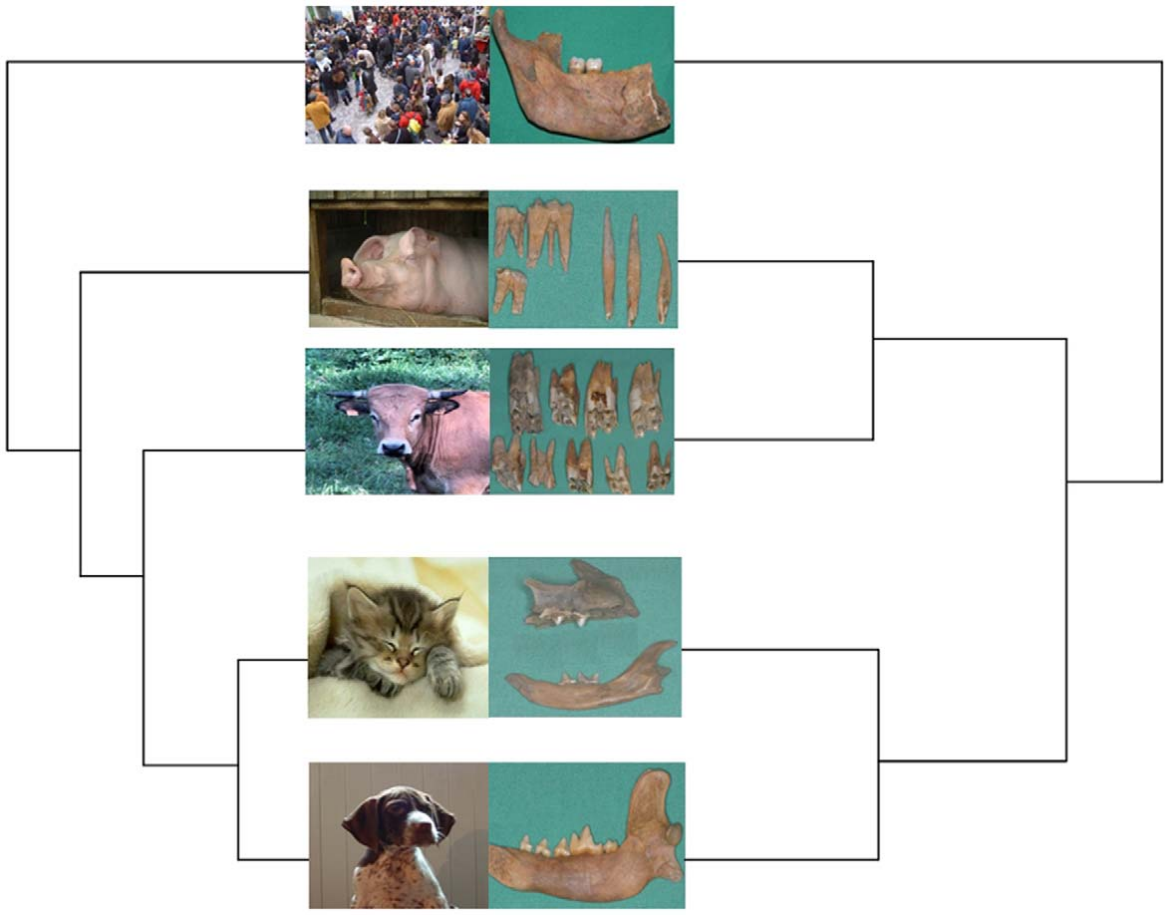
The dendrogram obtained by the software Maldi Biotyper 2.0 (Bruker Daltonics) after peptide spectral analysis is congruent with the one derived from cytochrome *b* sequencing by the software Tree View (Free down load from the Internet site: http://darwin.zoology.gla.ac.uk/~rpage/treeviewx/download.html **).** The images indicate the mammalian species (left) from which the ancient teeth were identified (right).

**Figure 2:**
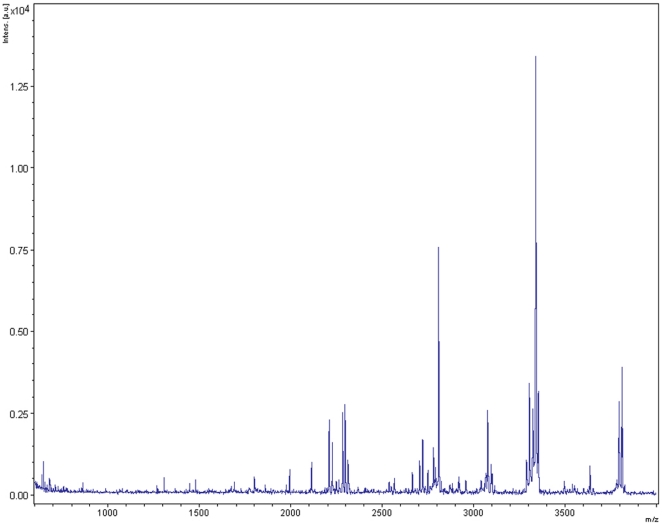
MALDI-TOF MS peptide spectrum obtained from 8,500-year-old human dental pulp.

We applied the same approach to ancient teeth. By using the software MALDI Biotyper 2.0 and comparing with our modern database, we could classify 10/18 (56%) teeth collected in 8/15 (53.3%) individuals from five burial sites dating between 700 and 8,500 years ago (Table S6). In these specimens, protein concentrations of 0.28±0.12 g/L yielded reproducible MALDI-TOF MS profiles that differed by 1–17 USSPs (Table S7). We did not observe contamination from one species to another one in this study. Peptide sequencing confirmed these data by giving 4 different proteins including collagen (alpha-1 type I and alpha-2 type I) in ancient human dental pulp (Table S4). Two isolated teeth collected from a first- or second-century site were identified as cow, four other teeth as dog and one additional tooth as cat. After these profiles of ancient individuals were incorporated into ancient mammal database, further comparison of teeth that had not been identified by first-round comparison with modern profiles yielded 100% identification with cow, cat, pig and human, with identification scores of≥1.8. All MALDI-TOF MS results were in agreement with the observed morphological characteristics and were confirmed by cytochrome *b* sequencing (Figure 1, Table S8). We therefore successfully identified the remains of individuals representing four domestic mammalian species frequently encountered in excavations. Two isolated teeth collected from an 18^th-^century site were identified as human, as were teeth collected from 300-year-old burial site in France. This approach also proved effective in classifying 8,500-year-old human dental pulp specimens collected in Syria from the deciduous teeth of two Neolithic children (Figure 2).

## Discussion

The results herein reported establish the proof-of-concept of accurate classification of mammal individuals by MALDI-TOF MS profiling of peptides prepared from the dental pulp. We used parallel cytochrome *b* gene sequencing reported in literature as a gold standard in molecular classification of mammals [3], [9] (Table S1) since the simple morphological examination of teeth may lack specificity.

A MALDI-TOF MS database has already been published for the identification of *Eucarya* species based on the analysis of cultured cell lines over 34 species ranging from insects to primates [5]. Moreover, MALDI-TOF MS was recently applied to the investigation of human circulating immune cells [13]. It was, however, necessary to build a new database because we analyzed an entire organ, mixing several tissues and cell lines. Indeed, unlike DNA sequencing, MALDI-TOF MS profiling relies on post-translational modifications, which may differ with tissue specificity. Also, we trypsin-digested the protein extracted from dental pulp prior to MALDI-TOF MS analysis, meaning we analyzed peptides instead of proteins. At last, peptide sequencing data also agreed with MALDI-TOF MS data which all indicated that the ancient dental pulp contained less numerous identifiable proteins than the modern dental pulp, probably resulting from the natural decay due to digenetic alteration. These data explained the necessity to complement the database of modern specimens by a database of ancient specimens in order to increase the ratio of classification of the ancient specimens.

We developed a simple protocol, with protein preparation being limited mainly to dialysis; indeed, we did not aim to identify unique proteins but instead to analyze a complex peptidic profile comprising several dozens of picks. Such identifying peptidic profiles were reproducible for any individual, and it was reproducible from one individual to another of the same species. Moreover, we observed that three USSPs herein reported have been already described in the case of human, rabbit and cow [7], thus validating our approach. This approach simplifies and strengthens previous approaches that rely on the analysis of a single soluble protein [10]. Testing of keratinous tissues (95% of the total material in feathers and down is composed of *α*-keratin) yielded four SEMPs and three USSPs for human hair, compared to 40 SEMPs and 17 USSPs from human dental pulp [10].

**Figure 3:**
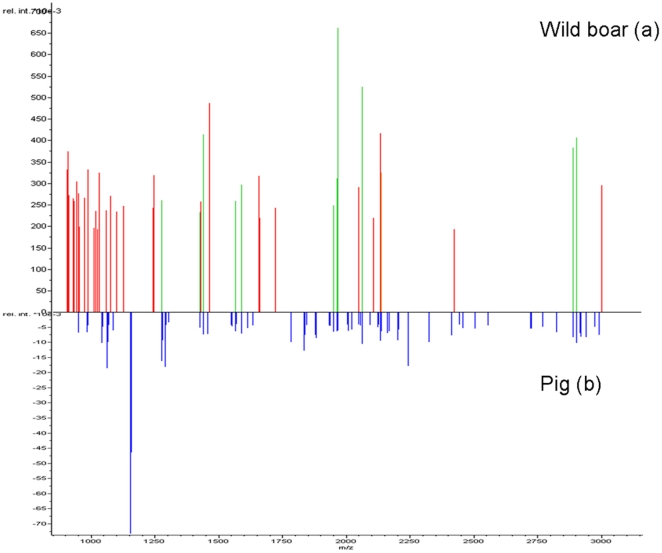
MALDI-TOF MS peptide profiling of proteins extracted from dental pulp. Pseudo-gel displays (Gel View, Bruker Daltonics) of the peptide spectra obtained with wild boar (a) and pig (b) specimens were used as the reference peptide. The mass-to-charge ratio (m/z) of each peptide is indicated on the x-axis, and the relative intensity of each peak is shown on the y-axis. Blue bars correspond to peaks from pig specimens, red bars correspond to peaks from wild boar specimens, and green bars represent peaks shared by both.

All MALDI-TOF MS results were in agreement with the observed morphological characteristics and were confirmed by cytochrome *b* sequencing (Figure 1, Table S1, Table S5, Table S6, Table S8). We therefore successfully classified the remains of individuals representing four domestic mammalian species frequently encountered in excavations. In addition, MALDI-TOF MS analysis of keratin recovered from the clothing of a 5,300-year-old Tyrolean mummy, which was remarkably preserved due to natural freezing in the Alps, revealed that the material originated from sheep and cattle [14]. The results presented here establish the proof-of-concept that MALDI-TOF MS peptide profiling of dental pulp can accurately classify ancient mammals at the species level. The finding that wild boars and pigs, which are members of the same genetic species, had 5 and 2 USSPs from a total of 11 and 12 SEMPs, respectively, illustrates the discriminatory power of MALDI-TOF MS peptide profiling (Figure 3). Ancient mammals were previously identified by analyzing dried muscle and skin from well-preserved museum specimens, hair shafts from permafrost-preserved animals, and bones and teeth of buried animals [15]–[18]. These tissues are directly exposed to the environment; contamination and degradation of surface proteins have been observed [19]. Reduced numbers of USSPs have been identified in tinted or bleached hair samples, and new peaks in these specimens have also been observed [10].

In contrast, dental pulp was our material of choice and this material has many advantages in studying ancient samples [20]. The tooth cavity protects the dental pulp from potential contamination and thermal degradation [21]. Moreover, teeth are the only body parts that remain after the bones have disintegrated. For instance, only teeth remained in the Early Upper Paleolithic site of Kostenski in Eastern Europe, which proved vital in understanding the dispersal of modern humans even though morphological identification was impossible [22]. In this study, we observed that the peptide spectra that were derived from the dental pulp contained several trypsin-digested proteins in addition to the collagen type I and collagen type III previously shown to be major constituents of the dental pulp [23]. We also found collagen type V - derived peaks, a collagen molecule also previously characterized in the dental pulp [23]. Peptide sequencing confirmed the detection of collagen in both modern and ancient dental pulp specimens, indicating that the dental pulp is a previously uninvestigated source for this protein which is widely used for the classification of ancient mammal individuals at the species level, in addition to bone tissue [7], [24]–[27]. Moreover, peptide sequencing still proved the detection of 2 additional proteins in ancient dental pulp specimens, which have been previously reported as present in the human dental pulp [23], [28], [29]. Sequencing therefore indicated that peptide profiling identification did not rely on only one protein and that the dental pulp is a suitable source of non-collagen proteins. In our hands, accurate identification was obtained within 72 hours. These results indicate that MALDI-TOF MS peptide profiling could be used for rapid classification at the species level of mammalian remains collected from excavations, thus valuably contributing to the study of past human and non-human populations.

### Conclusions

We observed that dental pulp samples were sufficient for both PCR-based sequence classification and MALDI-TOF MS peptide profiling in ancient and modern mammal individuals at the species level. Dental pulp is relatively easy to collect and is durable due to being embedded in solid teeth. Dental pulp is now potentially a new source for studying of ancient proteins, including collagen. Therefore, we recommend MALDI-TOF MS analysis of dental pulp as a reproducible approach complementing DNA sequencing for the accurate classification of forensic and ancient mammalian remains.

## Materials and Methods

### Sources of dental pulp

We analyzed 13 modern mammalian species (37 total individuals, 37 teeth): cow (*Bos taurus*), goat (*Capra hircus*), camel (*Camelus dromedarius*), wild boar (*Sus scrofa*), pig (*Sus scrofa* subsp. *domesticus*), roe deer (*Capreolus capreolus*), rabbit (*Oryctolagus cuniculus*), rat (*Rattus rattus*), guinea pig (*Cavia porcellus*), cat (*Felis catus*), dog (*Canis familiaris*), red fox (*Vulpes vulpes*) and human (*Homo sapiens*). Human teeth were acquired from dentists after obtaining informed written consent from the patients (Table S1). This study was approved by the Ethic Committee, Institute Fédératif de Recherche 48, Marseille, France. Ancient individuals were excavated from five archeological sites and initially classified by morphological criteria. The classified species included roman pig, cow and dog from the Lattes site (first or second century), France; cat from a 13^th^- or 14^th^-century site in Lille, France; and human teeth from a 6,500 BC site in Syria, a first- to third-century site in the Roman catacombs, Italy, and an 18^th-^century site in Douai, France. (Table S8). The criteria for the ancient teeth selected were used from previous publication [20].

### Nucleotide sequence–based classification

To confirm the classification of the collected samples, DNA was extracted from dental pulp, and a 307-bp partial cytochrome *b* sequence was amplified using primers L14841 and H15149 for modern samples [30]. Partial amplification (149 bp) of cytochrome *b* was performed in ancient samples using primers N2-cyt *b* F (5'-GTAGAATGAATCTGAGGCGG-3') and N2-cyt *b* R (5'-CCTGTAGGGTTGTTGGATCC-3'). DNA concentration was measured with a NanoDrop 1000 (Thermo Fisher Scientific, Courtaboeuf, France). Amplifications were performed in a 2720 Thermal Cycler (Applied Biosystems, Courtaboeuf, France) under the following conditions: activation for 10 min at 95°C, followed by 40 cycles of a 30-s denaturation step at 95°C, a 45-s hybridization step at 57°C and a 90-s elongation step at 72°C, with a final 7-min extension step at 72°C. The PCR mixtures consisted of 2.5 µL 10X PCR buffer, 2.5 µL dNTPs (2 mM each of dATP, dCTP, dGTP, dTTP), 0.8 µL MgCl_2_ (25 mM), 0.5 µL each primer (10 pmoL/ µL), 1.25 U HotStarTaq polymerase (Qiagen), 13.5 µL H_2_O, 0.5 µl BSA, and 4 µL template DNA. As a negative control, 4 µL distilled water was used in lieu of template DNA. PCR products were separated by electrophoresis for 26 min at 100 V in a 2% agarose gel with 0.5X TBE. PCR products were sequenced using a Big Dye Terminator kit and an ABI PRISM 3130 Genetic Analyzer (Applied BioSystems, Courtaboeuf, France). The sequences were analyzed using the ABI PRISM DNA Sequencing Analysis Software version 3.0 (Applied BioSystems) and compared to sequences available in the GenBank database using the BLAST algorithm (http://www.ncbi.nlm.nih.gov/blast/Blast.cgi).

### Sample preparation for MALDI-TOF peptide profiling

Total protein was extracted by incubating the dental pulp with 1 mL of 500 mM EDTA, pH 8.0, with agitation at room temperature for 24 hours. The samples were then sonicated five times for 1 min each and centrifuged at 17,900 x *g* for 40 min at room temperature. This was followed by dialysis overnight in 2 L of a solution containing 50 mM Tris-HCl, pH 8.0, and 150 mM NaCl. Protein concentration was determined using the Bradford protein quantification protocol. For MALDI-TOF analysis, 100 µL of the protein solution was digested overnight with 100 µL tryptic solution (12.5 ng/ µL trypsin, 50 mM NH_4_HCO_3_) at 30°C. Negative controls without specimen samples were run in parallel to test for contaminant peptides. The trypsin-digested peptides were purified with C18 ZipTips (Millipore, Billerica, MA, USA) following the manufacturer's instructions. An aliquot of 3 µL of the solution was spotted from the ZipTipC18 onto the MTP AnchorChip 384 TF MALDI steel plate. After each spot had dried, 1 µL of α-cyano-4-hydroxycinnamic acid matrix solution was applied on top of the sample. The samples were analyzed in an Autoflex II (MALDI-TOF, Bruker Daltonics GmbH, Leipzig, Germany) in reflectron mode. The mass list was derived automatically using FlexAnalysis software (Bruker Daltonics) with the following criteria: (*i*) SNR≥ 3, (*ii*) mass detection ranging from 700 to 4,000 Da and (*iii*) mass tolerance set to±0.5 Da. Calibration spectra were produced using the “user-defined” function of the FlexAnalysis software, set at 300 ppm. For each MALDI-TOF profile, we calculated the quality score by analyzing peaks between 900 and 3,000 Da. For each spectrum, we noted the number of peaks and the maximum value of the SNR. Two balanced parameters were taken into account to determine quality scores: (*i*) the number of peaks with a balance of 0 for n values≤8, 1 for 8<n≤27, 2 for 27<n ≤43, 3 for 43<n≤78, 4 for 78<n≤ 86, 5 for 86<n≤110, and 6 for n>110; and (*ii*) the maximal SNR with a balance of 0 for SNmax values≤6.8, 1 for 6.8<SNmax≤26.8, 2 for 26.8<SNmax≤52.6, 3 for 52.6<SNmax≤208.2, 4 for 208.2<SNmax≤398.6, 5 for 398.6<SNmax≤1,092.2, and 6 for SNmax>1,092.2. The quality scores  =  the value of the number of peaks + the value of the maximal SNR.

### MALDI-TOF MS classification

We observed contamination peaks in the negative controls, which were automatically removed from the spectra using the FlexAnalysis software (Bruker Daltonics). Manual annotation was necessary to eliminate some of the contaminant peaks that were not automatically discarded. A peptide spectrum database was then generated by analyzing dental pulp collected from 13 mammalian species, including human. An established public database for identifying the peptide spectra of the mammalian species used in our study could not be located. For each mammalian species, we analyzed at least two teeth collected from a minimum of two individuals. For each dental pulp specimen, two independent spots were analyzed with five independent acquisitions per spot, for a total of 10 spectra per dental pulp specimen. From 370 spectra (37×10 = 370), we selected the 279 spectra exhibiting quality scores superior 2 to contribute our modern mammal database by using the software Maldi Biotyper 2.0 (Bruker Daltonics). We analyzed peptides with molecular weights between 700 and 4,000 Da, resulting in a total of 42 to 207 peaks per specimen. We further established an in-silico trypsin digestion pattern of collagen molecules by retrieving collagen molecule protein sequences in http://www.uniprot.org/uniprot/P08123 and using the BioTools software for restriction pattern.

For blindly classification, we coded the protein samples from the same 37 teeth and applied the same approach MALDI-TOF MS. But for the blindly identification, we didn't need to remove the contamination peaks as we did for contributing the local modern database reference. 370 spectra from 37 coded protein samples were directly applied into the software MALDI Biotyper 2.0 for classification. A specimen was identified at the species level based on the highest identification score≥2.000 for modern mammal. In the case of ancient mammal, we decreased the classification score threshold because of the degradation of protein with time.

### MALDI Biotyper procedure of mammal species classification

The procedure of mammal species classification by using MALDI Biotyper 2.0 (Bruker Daltonics) was applied from the previously publication [31]. However, in this study, we edited processing methods suitable to our work: we analyzed from 900 to 3,000 Da (Figure S2).

### Peptide sequencing

Proteins extracted from one modern and two ancient human dental pulp specimens were separated by 1 D gel electrophoresis, bands were excised from the gel and digested with trypsin as described [32]. Tryptic peptides were analyzed by MS/MS on a nanoelectrospray ionization quadrupole TOF hybrid mass spectrometer (Q-TOF Ultima; Waters Micromass) coupled with a nano-HPLC (Cap-LC; Waters). The peptide and fragment ion masses obtained were matched automatically to proteins in a non-redundant database NCBI NR (version 20101105, taxonomy *Homo sapiens*, 232,072 sequences) using the Mascot version 2.3 MS/MS ions search algorithm (http://www.matrixscience.com). To validate protein identification, only matches with individual ion scores above 20 were considered.

## Supporting Information

Figure S1 In silico analysis of peptide spectrum derived from the human (*Homosapiens*) dental pulp.
(DOC)

Figure S2 Workflow of mammal individual classification at the species level using dental pulp MALDI-TOF MS peptide profiling.
(DOC)

Table S1 List of the modern dental pulp specimens used in the study.
(DOC)

Table S2 Observed mass to change ratio of semi-specific peaks (SEMPs) (1000–3000 Da) from 13 mammal species.
(DOC)

Table S3 The results of in-silico analysis of peptide spectra derived from the modern human (*Homo sapiens*) dental pulp, from the modern cow (*Bos taurus*) and the modern dog (*Canis familiaris*) dental pulp.
(DOC)

Table S4 Peptide sequencing of human dental pulp: “+” denotes the presence of the protein; “-”denotes the absence of the protein.
(DOC)

Table S5 Results of blindly classification of modern mammal individuals by MALDI-TOF MS.
(DOC)

Table S6 Results of classification of ancient mammal individuals by MALDI-TOF MS peptide profiling of the dental pulp.
(DOC)

Table S7 Observed mass to charge ratio of unique species-specific peaks (USSPs) (1000 – 3000 Da) from 13 mammal species. “m/z” values in bold characters have been reported by Buckley M. et al. [7].
(DOC)

Table S8 List of the ancient dental pulp specimens used in the study.
(DOC)
