# Supplementary material for: Classification of Ancient Mammal Individuals Using Dental Pulp MALDI-TOF MS Peptide Profiling
Source: PLoS One. 2011 Feb 25;6(2):e17319. doi: 10.1371/journal.pone.0017319 (PMC3045434; doi:10.1371/journal.pone.0017319)
Supplement: Figure S2 — Workflow of mammal individual classification at the species level using dental pulp MALDI-TOF MS peptide profiling. (DOC) [file pone.0017319.s002.doc]

Dental pulp

Protein extraction

Tryptic digestion

MALDI TOF MS/MS: AutoFlex

Analysis by FlexAnalysis

Remove peaks from blank sample

Analysis by MALDI BioTyper 2.0

Create MSP database

SEMPs

USSPs

Quality score

of spectrum

MSP modern

MSP ancient

Blind classification
