# Supplementary material for: Classification of Ancient Mammal Individuals Using Dental Pulp MALDI-TOF MS Peptide Profiling
Source: PLoS One. 2011 Feb 25;6(2):e17319. doi: 10.1371/journal.pone.0017319 (PMC3045434; doi:10.1371/journal.pone.0017319)
Supplement: Table S1 — List of the modern dental pulp specimens used in the study. (DOC) [file pone.0017319.s003.doc]

|  | **Mammal** | **Species** | **Individuals** | **Teeth** | **PCR cyt*b*** | |
| --- | --- | --- | --- | --- | --- | --- |
|  |  |  |  |  | **Sequencing** |  |
| **1** | **Human** | *Homo sapiens* | Woman 1 | F1D1 | *Homo sapiens* (100%) | AY509658 |
|  |  |  | Man 1 | H1D1 | *Homo sapiens* (100%) | AY509658 |
|  |  |  | Man 2 | H2D1 | *Homo sapiens* (100%) | AY509658 |
| **2** | **Rat** | *Rattus rattus* | Rat 36 | R36 | *Rattus rattus* (100%) | EF186475 |
|  |  |  | Rat 39 | R39 | *Rattus rattus* (100%) | EF186475 |
|  |  |  | Rat 40 | R40 | *Rattus rattus* (100%) | EF186475 |
| **3** | **Guinea-pig** | *Cavia porcellus* | Guinea pig 1 | GP1 | *Cavia porcellus* (100%) | AF490405 |
|  |  |  | Guinea pig 2 | GP2 | *Cavia porcellus* (100%) | AF490405 |
|  |  |  | Guinea pig 3 | GP3 | *Cavia porcellus* (100%) | AF490405 |
|  |  |  | Guinea pig 4 | GP4 | *Cavia porcellus* (100%) | AF490405 |
| **4** | **Rabbit** | *Oryctolagus cuniculus* | Rabbit 1 | RB1 | *Oryctolagus cuniculus* (100%) | AJ001588 |
|  |  |  | Rabbit 2 | RB2 | *Oryctolagus cuniculus* (100%) | AJ001588 |
|  |  |  | Rabbit 3 | RB3 | *Oryctolagus cuniculus* (100%) | AJ001588 |
| **5** | **Pig** | *Sus scrofa domesticus* | Pig 1 | P1 | *Sus scrofa* (100%) | FM205713 |
|  |  |  | Pig 2 | P2 | *Sus scrofa* (100%) | FM205713 |
| **6** | **Wild boar** | *Sus scrofa* | Wild boar 1 | WB1 | *Sus scrofa* (100%) | AF136549 |
|  |  |  | Wild boar 2 | WB2 | *Sus scrofa* (100%) | AF136549 |
|  |  |  | Wild boar 3 | WB3 | *Sus scrofa* (100%) | AF136549 |
| **7** | **Cow** | *Bos taurus* | Cow 1 | C1 | *Bos taurus* (100%) | EU365345 |
|  |  |  | Cow 2 | C2 | *Bos taurus* (100%) | EU365345 |
| **8** | **Goat** | *Capra hiscus* | Goat 1 | G1 | *Capra hiscus* (100%) | FM205715 |
|  |  |  | Goat 2 | G2 | *Capra hiscus* (100%) | FM205715 |
| **9** | **Roe deer** | *Capreolus capreolus* | Roe deer 1 | RD1 | *Capreolus capreolus* (100%) | AJ000024 |
|  |  |  | Roe deer 2 | RD2 | *Capreolus capreolus* (100%) | AJ000024 |
|  |  |  | Roe deer 3 | RD3 | *Capreolus capreolus* (100%) | AJ000024 |
| **10** | **Dromedary** | *Camelus dromedarius* | Dromedary 1 | D1 | *Camelus dromedarius* (100%) | AY126631 |
|  |  |  | Dromedary 2 | D2 | *Camelus dromedarius* (100%) | AY126631 |
|  |  |  | Dromedary 3 | D3 | *Camelus dromedarius* (100%) | AY126631 |
| **11** | **Dog** | *Canis familiaris* | Dog 1 | Dg1 | *Canis familiaris* (100%) | DQ309764 |
|  |  |  | Dog 2 | Dg2 | *Canis familiaris* (100%) | DQ309764 |
|  |  |  | Dog 3 | Dg3 | *Canis familiaris* (100%) | DQ309764 |
| **12** | **Red fox** | *Vulpes vulpes* | Red fox 1 | RF1 | *Vulpes vulpes* (100%) | DQ498124 |
|  |  |  | Red fox 2 | RF2 | *Vulpes vulpes* (100%) | DQ498124 |
|  |  |  | Red fox 3 | RF3 | *Vulpes vulpes* (100%) | DQ498124 |
|  |  |  | Red fox 4 | RF4 | *Vulpes vulpes* (100%) | DQ498124 |
| **13** | **Cat** | *Felis catus* | Cat 1 | Ca1 | *Felis catus* (100%) | AB194817 |
|  |  |  | Cat 2 | Ca2 | *Felis catus* (100%) | AB194817 |
