# Supplementary material for: Classification of Ancient Mammal Individuals Using Dental Pulp MALDI-TOF MS Peptide Profiling
Source: PLoS One. 2011 Feb 25;6(2):e17319. doi: 10.1371/journal.pone.0017319 (PMC3045434; doi:10.1371/journal.pone.0017319)
Supplement: Table S2 — Observed mass to change ratio of semi-specific peaks (SEMPs) (1000–3000 Da) from 13 mammal species. (DOC) [file pone.0017319.s004.doc]

|  | **COW** | | |
| --- | --- | --- | --- |
|  | **m/z** | **S/N** | **Delta m** |
| 1 | 1032,4211 | 3,25 ± 0,21 | 0,5 ± 0,03 |
| 2 | 1088,3531 | 3,57 ± 0,18 | 0,36 ± 0,01 |
| 3 | 1095,4479 | 5,7 ± 0,9 | 0,40 ± 0,01 |
| 4 | 1105,4887 | 22,25 ± 4,33 | 0,39 ± 0,01 |
| 5 | 1208,5856 | 19,67 ± 3,62 | 0,41 ± 0,01 |
| 6 | 1264,5358 | 6,5 ± 0,39 | 0,47 ± 0,02 |
| 7 | 1267,6053 | 7,63 ± 0,5 | 0,47 ± 0,02 |
| 8 | 1280,6518 | 6,93 ± 0,95 | 0,47 ± 0,04 |
| 9 | 1295,5792 | 5,36 ± 0,98 | 0,43 ± 0,03 |
| 10 | 1435,6447 | 27,84 ± 9,39 | 0,43 ± 0,01 |
| 11 | 1443,6726 | 16,41 ± 6,99 | 0,43 ± 0,02 |
| 12 | 1459,66 | 24,18 ± 5,29 | 0,42 ± 0,02 |
| 13 | 1473,6181 | 13,82 ± 5,39 | 0,44 ± 0,02 |
| 14 | 1532,7175 | 7,13 ± 2,11 | 0,44 ± 0,01 |
| 15 | 1586,739 | 17,5 ± 6,11 | 0,45 ± 0,01 |
| 16 | 1648,7907 | 12,87 ± 4,54 | 0,42 ± 0,02 |
| 17 | 1655,7819 | 7,12 ± 2,47 | 0,40 ± 0,02 |
| 18 | 1676,7804 | 8,59 ± 2,49 | 0,45 ± 0,02 |
| 19 | 1783,7755 | 6,24 ± 3,09 | 0,52 ± 0,06 |
| 20 | 1848,8523 | 18,53 ± 4,85 | 0,43 ± 0,03 |
| 21 | 1921,9168 | 10,37 ± 5,01 | 0,42 ± 0,05 |
| 22 | 1937,9593 | 25,7 ± 7,00 | 0,43 ± 0,03 |
| 23 | 1975,9957 | 24,25 ± 2,89 | 0,43 ± 0,03 |
| 24 | 1997,0015 | 21,09 ± 2,15 | 0,43 ± 0,03 |
| 25 | 2019,9858 | 13,46 ± 4,17 | 0,43 ± 0,03 |
| 26 | 2043,8226 | 7,61 ± 4,48 | 0,52 ± 0,03 |
| 27 | 2199,0449 | 11,18 ± 4,01 | 0,46 ± 0,04 |
| 28 | 2253,1452 | 16,33 ± 3,24 | 0,47 ± 0,03 |
| 29 | 2294,108 | 12,71 ± 4,18 | 0,46 ± 0,04 |
| 30 | 2309,0977 | 10,46 ± 4,55 | 0,48 ± 0,04 |
| 31 | 2410,2159 | 14,36 ± 4,23 | 0,48 ± 0,05 |
| 32 | 2418,2116 | 10,91 ± 5,11 | 0,51 ± 0,04 |
| 33 | 2487,2879 | 12,4 ± 4,79 | 0,49 ± 0,07 |
| 34 | 2513,2775 | 11,03 ± 5,39 | 0,50 ± 0,05 |
| 35 | 2565,303 | 9,5 ± 5,77 | 0,48 ± 0,05 |
| 36 | 2644,3584 | 9,3 ± 5,96 | 0,49 ± 0,06 |
| 37 | 2660,3436 | 7,35 ± 6,66 | 0,50 ± 0,07 |
| 38 | 2703,264 | 9,75 ± 6,39 | 0,61 ± 0,3 |
| 39 | 2719,3375 | 11,85 ± 6,06 | 0,51 ± 0,06 |
| 40 | 2735,378 | 10,07 ± 6,72 | 0,48 ± 0,07 |
| 41 | 2853,5012 | 11,84 ± 6,55 | 0,53 ± 0,06 |
| 42 | 2869,5217 | 19,65 ± 5,67 | 0,50 ± 0,07 |

|  | **CAT** | | |
| --- | --- | --- | --- |
|  | **m/z** | **S/N** | **Delta m** |
| 1 | 1105,4819 | 16,4 ± 4,67 | 0,40 ± 0,03 |
| 2 | 1409,731 | 16,56 ± 4,24 | 0,42 ± 0,03 |
| 3 | 1459,6161 | 12,96 ± 6,41 | 0,44 ± 0,04 |
| 4 | 1565,6834 | 4,28 ± 0,3 | 0,47 ± 0,04 |
| 5 | 1975,9085 | 7,77 ± 2,99 | 0,47 ± 0,03 |
| 6 | 2019,91 | 7,55 ± 1,04 | 0,48 ± 0,04 |
| 7 | 2052,993 | 7,1 ± 1,22 | 0,54 ± 0,03 |
| 8 | 2198,9268 | 7,57 ± 4,15 | 0,51 ± 0,04 |
| 9 | 2216,0137 | 7,51 ± 1,12 | 0,53 ± 0,03 |
| 10 | 2410,0995 | 5,42 ± 1,22 | 0,53 ± 0,04 |
| 11 | 2471,1682 | 4,58 ± 0,51 | 0,59 ± 0,04 |
| 12 | 2703,2172 | 7,27 ± 1,34 | 0,54 ± 0,04 |
| 13 | 2719,1695 | 7,39 ± 1,02 | 0,58 ± 0,02 |
| 14 | 2753,2787 | 5,6 ± 0,89 | 0,60 ±0,04 |
| 15 | 2853,3585 | 12,54 ± 2,56 | 0,58 ± 0,02 |
| 16 | 2869,3346 | 8,11 ± 2,80 | 0,60 ±0,05 |

|  |  | **GOAT** |  |
| --- | --- | --- | --- |
|  | **m/z** | **S/N** | **Delta m** |
| 1 | 1105,4952 | 20,51 ± 4,34 | 0,45 ± 0,02 |
| 2 | 1152,6321 | 7,09 ± 0,65 | 0,54 ± 0,03 |
| 3 | 1283,6326 | 26,79 ± 50,03 | 0,47 ± 0,02 |
| 4 | 1287,6754 | 4,73 ± 0,60 | 0,53 ± 0,03 |
| 5 | 1435,6636 | 19,69 ± 3,60 | 0,47 ± 0,02 |
| 6 | 1459,6718 | 14,56 ± 2,71 | 0,48 ± 0,03 |
| 7 | 1473,6649 | 6,65 ± 1,61 | 0,49 ± 0,03 |
| 8 | 1585,7541 | 15,9 ± 2,37 | 0,52 ± 0,03 |
| 9 | 1586,8267 | 4,79 ± 2,26 | 0,56 ± 0,14 |
| 10 | 1648,8555 | 18,34 ± 1,79 | 0,49 ± 0,02 |
| 11 | 1743,8433 | 4,01 ± 0,53 | 0,57 ± 0,04 |
| 12 | 1779,9525 | 5,01 ± 0,85 | 0,54 ± 0,09 |
| 13 | 1821,8941 | 4,14 ± 0,67 | 0,60 ± 0,06 |
| 14 | 1848,8849 | 6,01 ± 1,24 | 0,522 ± 0,06 |
| 15 | 1931,0892 | 5,54 ± 0,83 | 0,61 ± 0,04 |
| 16 | 1976,0518 | 15,48 ± 1,31 | 0,51 ± 0,03 |
| 17 | 2020,0432 | 12,90 ± 1,70 | 0,53 ± 0,03 |
| 18 | 2089,0911 | 4,01 ± 0,36 | 0,56 ± 0,05 |
| 19 | 2105,0891 | 5,95 ± 0,64 | 0,53 ± 0,04 |
| 20 | 2199,0823 | 8,71 ± 0,91 | 0,54 ± 0,03 |
| 21 | 2232,1598 | 8,75 ± 1,38 | 0,52 ± 0,03 |
| 22 | 2409,233 | 12,45 ± 3,27 | 0,55 ± 0,04 |
| 23 | 2513,3102 | 14,75 ± 2,13 | 0,56 ± 0,03 |
| 24 | 2565,3132 | 9,49 ± 1,24 | 0,53 ± 0,04 |
| 25 | 2718,337 | 5,86 ± 1,08 | 0,57 ± 0,04 |
| 26 | 2766,393 | 7,63 ± 0,80 | 0,57 ± 0,05 |
| 27 | 2939,6513 | 6,21 ± 1,91 | 0,78 ± 0,31 |

|  | **DOG** | | |
| --- | --- | --- | --- |
|  | **m/z** | **S/N** | **Delta m** |
| 1 | 1076,439 | 4,93 ± 1,13 | 0,51 ± 0,05 |
| 2 | 1105,4702 | 19,8 ± 3,93 | 0,43 ± 0,03 |
| 3 | 1164,3948 | 5,34 ± 1,40 | 0,48 ± 0,06 |
| 4 | 1261,523 | 5,09 ± 0,85 | 0,49 ± 0,04 |
| 5 | 1459,6443 | 15,01 ± 2,46 | 0,49 ± 0,04 |
| 6 | 1469,7479 | 30,59 ± 6,10 | 0,49 ± 0,04 |
| 7 | 1473,6046 | 6,08 ± 1,09 | 0,50 ± 0,04 |
| 8 | 1566,7114 | 5,30 ± 0,97 | 0,51 ± 0,04 |
| 9 | 1576,7751 | 7,65 ± 1,51 | 0,51 ± 0,05 |
| 10 | 1586,726 | 10,8 ± 2,54 | 0,52 ± 0,05 |
| 11 | 1590,7751 | 7,91 ± 2,28 | 0,56 ± 0,06 |
| 12 | 1743,7845 | 32,14 ± 5,29 | 0,52 ± 0,04 |
| 13 | 1931,0999 | 14,31 ± 3,97 | 0,61 ± 0,07 |
| 14 | 1953,1315 | 6,72 ± 1,55 | 0,65 ± 0,04 |
| 15 | 1992,1264 | 31,73 ± 4,78 | 0,64 ± 0,05 |
| 16 | 2019,9884 | 10,35 ± 1,54 | 0,54 ± 0,04 |
| 17 | 2046,072 | 11,29 ± 1,69 | 0,59 ± 0,05 |
| 18 | 2105,0316 | 8,55 ± 1,43 | 0,61 ± 0,06 |
| 19 | 2194,0463 | 4,89 ± 0,76 | 0,63 ± 0,04 |
| 20 | 2198,9959 | 12,08 ± 1,84 | 0,59 ± 0,05 |
| 21 | 2216,0556 | 4,33 ± 0,57 | 0,65 ± 0,05 |
| 22 | 2261,2322 | 3,78 ± 0,52 | 0,62 ± 0,03 |
| 23 | 2271,3189 | 4,14 ± 0,67 | 0,68 ± 0,05 |
| 24 | 2330,1612 | 4,69 ± 0,81 | 0,71 ± 0,08 |
| 25 | 2350,1781 | 23,29 ± 3,21 | 0,57 ± 0,06 |
| 26 | 2497,2595 | 3,64 ± 0,30 | 0,63 ± 0,05 |
| 27 | 2785,3648 | 3,79 ± 0,38 | 0,66 ± 0,06 |
| 28 | 2820,4338 | 5,03 ± 0,75 | 0,68 ± 0,08 |
| 29 | 2847,4481 | 56,14 ± 14,88 | 0,64 ± 0,06 |
| 30 | 2853,4674 | 15,36 ± 16,79 | 0,63 ± 0,07 |
| 31 | 2869,4663 | 14,07 ± 1,98 | 0,63 ± 0,07 |
| 32 | 2911,2221 | 8,26 ± 2,84 | 1,14 ± 0,53 |
| 33 | 2975,4311 | 5,62 ± 0,68 | 0,71 ± 0,11 |
| 34 | 2999,5726 | 8,04 ± 1,03 | 0,67 ± 0,09 |

|  | **ROE DEER** | | |
| --- | --- | --- | --- |
|  | **m/z** | **S/N** | **Delta m** |
| 1 | 1060,5119 | 9,79 ± 2,19 | 0,05 ± 0,04 |
| 2 | 1105,4778 | 7,92 ± 2,43 | 0,47 ± 0,03 |
| 3 | 1130,5226 | 10,94 ± 1,49 | 0,48 ± 0,03 |
| 4 | 1152,6769 | 69,12 ± 9,23 | 0,48 ± 0,03 |
| 5 | 1161,5632 | 6,95 ± 0,80 | 0,61 ± 0,04 |
| 6 | 1163,4982 | 8,29 ± 1,31 | 0,46 ± 0,07 |
| 7 | 1265,789 | 365,14 ± 39,36 | 0,51 ± 0,03 |
| 8 | 1283,6272 | 5,73 ± 0,98 | 0,53 ± 0,04 |
| 9 | 1287,671 | 13,04 ± 2,69 | 0,52 ± 0,03 |
| 10 | 1309,6568 | 8,64 ± 1,62 | 0,58 ± 0,03 |
| 11 | 1459,6414 | 5,47 ± 1,35 | 0,54 ± 0,04 |
| 12 | 1611,7162 | 4,78 ± 0,66 | 0,66 ± 0,05 |
| 13 | 1648,7969 | 5,88 ± 0,79 | 0,59 ± 0,03 |
| 14 | 2338,1745 | 5,30 ± 1,43 | 0,60 ± 0,07 |
| 15 | 2438,2798 | 10,41 ± 1,58 | 0,61 ± 0,03 |
| 16 | 2533,3686 | 4,35 ± 0,49 | 0,65 ± 0,05 |
| 17 | 2757,4638 | 6,97 ± 0,92 | 0,67 ± 0,03 |
| 18 | 2870,5746 | 59,07 ± 7,94 | 0,61 ± 0,03 |
| 19 | 2983,6772 | 123,53 ± 15,16 | 0,60 ± 0,02 |

|  | **GUINEA-PIG** | | |
| --- | --- | --- | --- |
|  | **m/z** | **S/N** | **Delta m** |
| 1 | 1105,3159 | 7,30 ± 1,29 | 0,46 ± 0,04 |
| 2 | 1435,4445 | 7,32 ± 1,53 | 0,50 ± 0,05 |
| 3 | 1471,4977 | 5,05 ± 0,38 | 0,64 ± 0,13 |
| 4 | 1501,4659 | 6,88 ± 1,45 | 0,54 ± 0,05 |
| 5 | 1586,475 | 4,93 ± 0,94 | 0,57 ± 0,05 |
| 6 | 1743,5634 | 33,55 ± 5,54 | 0,51 ± 0,04 |
| 7 | 1757,5872 | 5,18 ± 0,67 | 0,58 ± 0,06 |
| 8 | 1772,5985 | 8,45 ± 1,95 | 0,52 ± 0,04 |
| 9 | 1930,8638 | 14,80 ± 2,42 | 0,53 ± 0,05 |
| 10 | 1945,7224 | 10,46 ± 0,95 | 0,56 ± 0,04 |
| 11 | 1998,7276 | 4,04 ± 0,58 | 1,36 ± 0,39 |
| 12 | 2014,6332 | 13,73 ± 2,92 | 0,53 ± 0,03 |
| 13 | 2017,803 | 11,49 ± 2,04 | 0,69 ± 0,39 |
| 14 | 2087,8499 | 4,10 ± 0,54 | 0,68 ± 0,06 |
| 15 | 2100,8085 | 5,42 ± 1,04 | 0,59 ± 0,04 |
| 16 | 2103,8787 | 13,20 ± 1,04 | 0,57 ± 0,04 |
| 17 | 2124,7526 | 4,95 ± 0,77 | 0,78 ± 0,31 |
| 18 | 2267,9535 | 20,00 ± 2,36 | 0,56 ± 0,03 |
| 19 | 2423,011 | 5,74 ± 0,54 | 0,66 ± 0,05 |
| 20 | 2767,0372 | 4,79 ± 0,83 | 0,61 ± 0,05 |
| 21 | 2899,1125 | 8,94 ± 1,61 | 0,62 ± 0,04 |
| 22 | 2915,2734 | 62,26 ± 26,86 | 0,63 ± 0,06 |
| 23 | 2933,2407 | 24,70 ± 7,79 | 0,62 ± 0,06 |

|  |  | **RAT** |  |
| --- | --- | --- | --- |
|  | **m/z** | **S/N** | **Delta m** |
| 1 | 1051,5195 | 6,14 ± 1,18 | 0,49 ± 0,06 |
| 2 | 1105,4939 | 17,93 ± 2,29 | 0,45 ± 0,04 |
| 3 | 1451,6588 | 4,52 ± 0,44 | 0,56 ± 0,04 |
| 4 | 1572,6691 | 5,33 ± 0,67 | 0,61 ± 0,07 |
| 5 | 1655,7644 | 6,25 ± 0,46 | 0,55 ± 0,05 |
| 6 | 1671,7782 | 5,1 ± 0,57 | 0,55 ± 0,06 |
| 7 | 1840,8591 | 4,59 ± 0,73 | 0,56 ± 0,07 |
| 8 | 1975,9343 | 8,35 ± 0,88 | 0,59 ± 0,07 |
| 9 | 2014,9626 | 17,4 ± 1,76 | 0,57 ± 0,05 |
| 10 | 2081,9776 | 5,05 ± 0,46 | 0,65 ± 0,07 |
| 11 | 2098,0493 | 4,22 ± 0,48 | 0,65 ± 0,08 |
| 12 | 2277,1019 | 4,70 ± 0,65 | 0,61 ± 0,07 |
| 13 | 2471,2275 | 5,96 ± 0,70 | 0,64 ± 0,09 |
| 14 | 2679,2954 | 5,20 ± 0,59 | 0,62 ± 0,07 |
| 15 | 2695,2961 | 10,73 ± 1,09 | 0,61 ± 0,06 |

|  | **RED-FOX** | | |
| --- | --- | --- | --- |
|  | **m/z** | **S/N** | **Delta m** |
| 1 | 1105,4825 | 11,51 ± 1,75 | 0,44 ± 0,02 |
| 2 | 1437,6805 | 11,80 ± 1,22 | 0,49 ± 0,02 |
| 3 | 1459,6456 | 8,26 ± 1,25 | 0,50 ± 0,02 |
| 4 | 1469,7071 | 7,40 ± 0,81 | 0,49 ± 0,02 |
| 5 | 1565,7361 | 3,71 ± 0,44 | 0,59 ± 0,08 |
| 6 | 1655,7488 | 3,84 ± 0,52 | 0,53 ± 0,04 |
| 7 | 1967,9148 | 4,20 ± 0,31 | 0,58 ± 0,02 |
| 8 | 1992,1908 | 58,24 ± 5,39 | 0,53 ± 0,03 |
| 9 | 2012,9696 | 5,9 ± 0,52 | 0,51 ± 0,03 |
| 10 | 2019,9618 | 5,86 ± 0,73 | 0,52 ± 0,03 |
| 11 | 2027,1134 | 8,42 ± 0,72 | 0,54 ± 0,03 |
| 12 | 2104,0505 | 9,02 ± 1,01 | 0,55 ± 0,04 |
| 13 | 2198,9672 | 4,31 ± 0,55 | 0,57 ± 0,07 |
| 14 | 2261,2679 | 8,13 ± 1,15 | 0,57 ± 0,03 |
| 15 | 2312,156 | 6,31 ± 0,86 | 0,55 ± 0,05 |
| 16 | 2350,219 | 30,45 ± 3,06 | 0,53 ± 0,03 |
| 17 | 2486,2455 | 5,67 ± 0,50 | 0,56 ± 0,07 |
| 18 | 2719,2766 | 4,81 ± 0,59 | 0,62 ± 0,11 |
| 19 | 2737,4841 | 4,31 ± 0,58 | 0,61 ± 0,09 |
| 20 | 2785,4201 | 6,71 ± 0,87 | 0,64 ± 0,07 |
| 21 | 2847,4149 | 29,51 ± 3,28 | 0,58 ± 0,05 |
| 22 | 2853,4495 | 8,78 ± 1,13 | 0,57 ± 0,03 |
| 23 | 2869,433 | 12,14 ± 1,34 | 0,58 ± 0,04 |
| 24 | 2911,3189 | 26,69 ± 3,64 | 0,57 ± 0,04 |
| 25 | 2957,6348 | 16,20 ± 1,52 | 0,61 ± 0,04 |
| 26 | 2975,3969 | 5,09 ± 0,67 | 0,64 ± 0,08 |
| 27 | 2999,5273 | 9,83 ± 1,17 | 0,61 ± 0,07 |

|  | **CAMEL** | | |
| --- | --- | --- | --- |
|  | **m/z** | **S/N** | **Delat m** |
| 1 | 1105,3464 | 37,3 ± 11,10 | 0,38 ± 0,05 |
| 2 | 1161,3947 | 3,58 ± 0,41 | 0,54 ± 0,05 |
| 3 | 1221,4563 | 6,63 ± 1,34 | 0,48 ± 0,05 |
| 4 | 1435,5666 | 22,88 ± 5,10 | 0,41 ± 0,04 |
| 5 | 1459,5876 | 19,89 ± 4,46 | 0,41 ± 0,04 |
| 6 | 1469,7191 | 4,43 ± 0,82 | 0,59 ± 0,08 |
| 7 | 1473,5706 | 8,41 ± 1,25 | 0,45 ± 0,04 |
| 8 | 1496,589 | 4,77 ± 0,64 | 0,43 ± 0,05 |
| 9 | 1550,6737 | 8,52 ± 1,74 | 0,41 ± 0,02 |
| 10 | 1586,6964 | 14,96 ± 4,57 | 0,43 ± 0,03 |
| 11 | 1634,7595 | 6,32 ± 1,05 | 0,44 ± 0,04 |
| 12 | 1655,7541 | 8,10 ± 2,06 | 0,40 ± 0,02 |
| 13 | 1790,8862 | 6,73 ± 1,41 | 0,47 ± 0,04 |
| 14 | 1961,9612 | 6,65 ± 2,09 | 0,64 ± 0,05 |
| 15 | 1976,0538 | 31,91 ± 8,44 | 0,46 ± 0,03 |
| 16 | 2006,034 | 10,18 ± 1,75 | 0,46 ± 0,04 |
| 17 | 2043,0622 | 11,12 ± 3,86 | 0,72 ± 0,06 |
| 18 | 2199,1588 | 6,59 ± 1,32 | 0,49 ± 0,05 |
| 19 | 2410,3397 | 10,28 ± 2,99 | 0,51 ± 0,05 |
| 20 | 2454,3228 | 7,60 ± 1,92 | 0,55 ± 0,05 |
| 21 | 2471,4173 | 7,20 ± 1,62 | 0,56 ± 0,06 |
| 22 | 2513,4077 | 7,64 ± 1,19 | 0,56 ± 0,05 |
| 23 | 2704,9057 | 16,44 ± 5,06 | 0,69 ± 0,40 |
| 24 | 2719,4889 | 13,85 ± 4,69 | 0,68 ± 0,27 |
| 25 | 2727,5048 | 8,44 ± 1,73 | 0,82 ± 0,27 |
| 26 | 2741,5661 | 14,24 ± 3,20 | 0,57 ± 0,06 |
| 27 | 2959,7381 | 16,35 ±6,67 | 0,60 ± 0,10 |

|  | **WILD BOAR** | | |
| --- | --- | --- | --- |
|  | **m/z** | **S/N** | **Delta m** |
| 1 | 1105,4757 | 4,61 ± 0,67 | 0,41 ± 0,04 |
| 2 | 1198,5802 | 3,85 ± 0,20 | 0,44 ± 0,06 |
| 3 | 1240,5751 | 4,08 ± 0,47 | 0,45 ± 0,05 |
| 4 | 1296,6194 | 15,26 ± 0,61 | 0,44 ± 0,04 |
| 5 | 1422,6603 | 3,95 ± 0,40 | 0,44 ± 0,01 |
| 6 | 1445,6788 | 4,65 ± 0,55 | 0,47 ± 0,06 |
| 7 | 1459,6576 | 7,98 ± 0,77 | 0,46 ± 0,03 |
| 8 | 1655,7809 | 4,50 ± 0,35 | 0,49 ± 0,05 |
| 9 | 1961,9912 | 5,79 ± 0,59 | 0,52 ± 0,05 |
| 10 | 2013,0061 | 4,45 ± 0,55 | 0,49 ± 0,06 |
| 11 | 2110,0825 | 5,63 ± 1,54 | 0,52 ± 0,07 |

|  | **PIG** | | |
| --- | --- | --- | --- |
|  | **m/z** | **S/N** | **Delta m** |
| 1 | 1105,4436 | 11,40 ± 1,94 | 0,39 ± 0,04 |
| 2 | 1121,4685 | 4,83 ± 0,80 | 0,35 ± 0,05 |
| 3 | 1240,5708 | 15,70 ± 2,95 | 0,40 ± 0,05 |
| 4 | 1459,6215 | 18,51 ± 3,74 | 0,43 ± 0,04 |
| 5 | 1550,7333 | 6,07 ± 1,88 | 0,52 ± 0,07 |
| 6 | 1655,773 | 6,19 ± 1,31 | 0,48 ± 0,07 |
| 7 | 1934,1196 | 5,81 ± 0,91 | 0,60 ± 0,05 |
| 8 | 1961,9572 | 18,7 ± 4,10 | 0,51 ± 0,05 |
| 9 | 2012,9817 | 8,17 ± 1,69 | 0,53 ± 0,07 |
| 10 | 2027,0281 | 4,23 ± 0,60 | 0,57 ± 0,07 |
| 11 | 2274,1078 | 5,87 ± 1,39 | 0,54 ± 0,05 |
| 12 | 2549,2212 | 5,53 ± 1,34 | 0,74 ± 0,22 |

|  | **RABBIT** | | |
| --- | --- | --- | --- |
|  | **m/z** | **S/N** | **Delta m** |
| 1 | 1083,488 | 5,75 ± 0,60 | 0,40 ± 0,02 |
| 2 | 1105,4417 | 5,56 ± 0,53 | 0,40 ± 0,02 |
| 3 | 1191,4568 | 3,51 ± 0,68 | 0,53 ± 0,04 |
| 4 | 1261,5512 | 5,41 ± 0,47 | 0,39 ± 0,02 |
| 5 | 1277,5677 | 24,3 ± 3,11 | 0,40 ± 0,02 |
| 6 | 1291,6095 | 17,24 ± 2,53 | 0,40 ± 0,02 |
| 7 | 1353,8018 | 5,81 ± 1,23 | 0,59 ± 0,04 |
| 8 | 1467,7551 | 21,53 ± 1,71 | 0,41 ± 0,02 |
| 9 | 1476,6832 | 10,99 ± 0,69 | 0,42 ± 0,02 |
| 10 | 1501,6799 | 6,60 ± 0,63 | 0,49 ± 0,02 |
| 11 | 1583,8355 | 5,50 ± 0,69 | 0,53 ± 0,04 |
| 12 | 1860,984 | 4,05 ± 0,80 | 0,47 ± 0,05 |
| 13 | 1947,9491 | 7,32 ± 0,86 | 0,43 ± 0,05 |
| 14 | 2005,9523 | 5,99 ± 0,63 | 0,45 ± 0,04 |
| 15 | 2027,0162 | 4,32 ± 0,38 | 0,52 ± 0,06 |
| 16 | 2059,0714 | 11,43 ± 2,83 | 0,40 ± 0,04 |
| 17 | 2129,1316 | 9,25 ± 1,27 | 0,47 ± 0,04 |
| 18 | 2193,2125 | 93,38 ± 16,15 | 0,47 ± 0,04 |
| 19 | 2513,2476 | 5,02 ± 0,56 | 0,51 ± 0,04 |
| 20 | 2530,2685 | 10,96 ± 1,85 | 0,51 ± 0,03 |
| 21 | 2697,4184 | 24,83 ± 5,95 | 0,52 ± 0,05 |
| 22 | 2719,3218 | 8,66 ± 1,24 | 0,59 ± 0,05 |
| 23 | 2727,3754 | 5,29 ± 0,57 | 0,58 ± 0,06 |
| 24 | 2763,366 | 6,15 ± 0,86 | 0,54 ± 0,07 |
| 25 | 2785,5103 | 4,30 ± 0,59 | 0,56 ± 0,05 |
| 26 | 2847,4369 | 13,23 ± 2,34 | 0,54 ± 0,05 |
| 27 | 2953,6149 | 16,41 ± 3,04 | 0,52 ± 0,05 |
| 28 | 2959,4107 | 5,11 ± 0,72 | 0,54 ± 0,05 |
| 29 | 2975,5653 | 7,06 ± 1,01 | 0,58 ± 0,05 |

|  | **HUMAN** | | |
| --- | --- | --- | --- |
|  | **m/z** | **S/N** | **Delta m** |
| 1 | 1105,4691 | 11,36 ± 4,82 | 0,33 ± 0,03 |
| 2 | 1189,4838 | 22,54 ± 13,7 | 0,33 ± 0,03 |
| 3 | 1235,5162 | 4,42 ± 1,15 | 0,39 ± 0,02 |
| 4 | 1454,2891 | 5,82 ± 3,43 | 0,50 ± 0,07 |
| 5 | 1459,5939 | 9,38 ± 4,38 | 0,33 ± 0,03 |
| 6 | 1467,7537 | 95,32 ± 26,22 | 0,32 ± 0,03 |
| 7 | 1493,6722 | 13,54 ± 5,40 | 0,31 ± 0,02 |
| 8 | 1533,6058 | 3,84 ± 1,63 | 0,34 ± 0,06 |
| 9 | 1546,7168 | 15,14 ± 7,75 | 0,32 ± 0,03 |
| 10 | 1580,6741 | 7,26 ± 2,62 | 0,34 ± 0,07 |
| 11 | 1586,6692 | 10,28 ± 4,30 | 0,36 ± 0,11 |
| 12 | 1623,7253 | 35,58 ± 12,60 | 0,31 ± 0,02 |
| 13 | 1699,7733 | 4,00 ± 1,11 | 0,34 ± 0,04 |
| 14 | 1742,7811 | 7,86 ± 1,72 | 0,32 ± 0,04 |
| 15 | 1812,8048 | 5,84 ± 2,26 | 0,30 ± 0,06 |
| 16 | 1848,8149 | 8,80 ± 5,40 | 0,31 ± 0,07 |
| 17 | 1898,9629 | 86,66 ± 34,18 | 0,31 ± 0,04 |
| 18 | 1961,9419 | 16,68 ± 10,56 | 0,30 ± 0,05 |
| 19 | 2003,953 | 14,82 ± 9,54 | 0,30 ± 0,06 |
| 20 | 2027,9949 | 6,10 ± 1,62 | 0,36 ± 0,09 |
| 21 | 2062,0375 | 11,34 ± 3,21 | 0,33 ± 0,07 |
| 22 | 2081,0111 | 40,04 ± 11,89 | 0,31 ± 0,06 |
| 23 | 2090,0478 | 8,02 ± 2,67 | 0,35 ± 0,05 |
| 24 | 2096,9947 | 4,98 ± 0,51 | 0,33 ± 0,10 |
| 25 | 2104,9879 | 6,32 ± 3,05 | 0,36 ± 0,11 |
| 26 | 2120,9851 | 6,98 ± 3,70 | 0,30 ± 0,03 |
| 27 | 2188,0805 | 5,06 ± 1,30 | 0,33 ± 0,07 |
| 28 | 2198,9741 | 7,40 ± 5,46 | 0,41 ± 0,18 |
| 29 | 2232,0813 | 7,56 ± 3,49 | 0,37 ± 0,09 |
| 30 | 2265,1099 | 10,06 ± 3,43 | 0,34 ± 0,09 |
| 31 | 2281,1185 | 5,22 ± 2,27 | 0,42 ± 0,11 |
| 32 | 2284,1348 | 12,44 ± 7,69 | 0,38 ± 0,14 |
| 33 | 2410,1529 | 11,18 ± 6,11 | 0,39 ± 0,11 |
| 34 | 2513,2431 | 11,40 ± 7,02 | 0,43 ± 0,16 |
| 35 | 2801,4092 | 5,46 ± 4,03 | 0,52 ± 0,15 |
| 36 | 2833,4493 | 8,38 ± 5,84 | 0,51 ± 0,10 |
| 37 | 2847,4426 | 19,68 ± 4,58 | 0,53 ± 0,12 |
| 38 | 2885,5042 | 17,22 ± 10,92 | 0,52 ± 0,12 |
| 39 | 2957,5294 | 17,44 ± 13,35 | 0,61 ± 0,16 |
| 40 | 2960,487 | 11,56 ± 9,83 | 0,48 ± 0,05 |
