# Supplementary material for: Classification of Ancient Mammal Individuals Using Dental Pulp MALDI-TOF MS Peptide Profiling
Source: PLoS One. 2011 Feb 25;6(2):e17319. doi: 10.1371/journal.pone.0017319 (PMC3045434; doi:10.1371/journal.pone.0017319)
Supplement: Table S3 — The results of in-silico analysis of peptide spectra derived from the modern human (Homo sapiens) dental pulp, from the modern cow (Bos taurus) and the modern dog (Canis familiaris) dental pulp. (DOC) [file pone.0017319.s005.doc]

| Human (*Homo sapiens*) | Tooth 1 - Women | Tooth 2 - Women | Tooth 1 - Men 1 | Tooth 2 - Men 1 | Tooth 1 - Men 2 | Tooth 2 - Men 2 |
| --- | --- | --- | --- | --- | --- | --- |
| Collagen alpha-1(I) chain OS=Homo sapiens | 12.4% | 12.7% | 14.9% | 11.7% | 15.4% | 10.3% |
| Collagen alpha-2(I) chain OS=Homo sapiens | 9.8% | 6.3% | 9.5% | 10.8% | 11.4% | 5.8% |
| Collagen alpha-1(III) chain OS=Homo sapiens | 5.7% | 7.6% | 8.9% | 10% | 10.6% | 7.7% |
| Collagen alpha-1(V) chain OS=Homo sapiens | 10.7% | 10.1% | 11.9% | 13.3% | 13% | 10.3% |
| Collagen alpha-2(V) chain OS=Homo sapiens | 4.9% | 5.1% | 3.7% | 5.8% | 5.7% | 5.2% |
| Collagen alpha-3(V) chain OS=Homo sapiens | 8.2% | 6.3% | 8.2% | 9.2% | 8.9% | 7.7% |
|  |  |  |  |  |  |  |
| Cow (*Bos taurus*) | Cow 1 | Cow 2 |  |  |  |  |
| Collagen alpha-1(I) chain OS=Bos taurus | 6.3% | 6% |  |  |  |  |
| Collagen alpha-1(III) chain OS=Bos taurus | 4.7% | 4.3% |  |  |  |  |
|  |  |  |  |  |  |  |
| Dog (*Canis familiaris*) | Dog 1 | Dog 2 | Dog 3 |  |  |  |
| Collagen alpha-1(I) chain OS=Canis familiaris | 9.0% | 11.1% | 9.2% |  |  |  |
