# Supplementary material for: Classification of Ancient Mammal Individuals Using Dental Pulp MALDI-TOF MS Peptide Profiling
Source: PLoS One. 2011 Feb 25;6(2):e17319. doi: 10.1371/journal.pone.0017319 (PMC3045434; doi:10.1371/journal.pone.0017319)
Supplement: Table S4 — Peptide sequencing of human dental pulp: “+” denotes the presence of the protein; “-”denotes the absence of the protein. (DOC) [file pone.0017319.s006.doc]

| **Protein** | **Modern tooth** | **Ancient teeth** | **References** |
| --- | --- | --- | --- |
| Serum albumin [Homo sapiens] | + | - | [29] |
| prepro-alpha1(I) collagen [Homo sapiens] | + | + (low score) | [23, 33] |
| Chain E, The Isomorphous Structures Of Prethrombin2, Hirugen-And Ppack-Thrombin | + | - |  |
| RecName: Full=Alpha-2-HS-glycoprotein; | + | - | [23] |
| pre-pro-alpha-2 type I collagen [Homo sapiens] | + | + (low score) | [23, 33] |
| ALB protein [Homo sapiens] (albumin) | + | - | [29] |
| antithrombin III [Homo sapiens] | + | - |  |
| Chain L, Structure Of The Hirulog 3-Thrombin Complex | + | + |  |
| Chain A, Human Serum Albumin In A Complex With Myristic Acid And Tri- Iodobenzoic Acid | + | - | [29] |
| RecName: Full=Alpha-1-acid glycoprotein 1; | + | - | [23] |
| unnamed protein product [Homo sapiens] (Vitronectin [Homo sapiens]) | + | - |  |
| biglycan [Homo sapiens] | + | - | [34] |
| histone H1.2 [Homo sapiens] | + | - |  |
| serum albumin precursor [Homo sapiens] | + | - | [29] |
| alpha-1-antitrypsin (aa 268-394) [Homo sapiens] | + | - |  |
| dermcidin preproprotein [Homo sapiens] | + | - |  |
| coagulation factor X precursor [Homo sapiens] |  | + |  |
