# Supplementary material for: Classification of Ancient Mammal Individuals Using Dental Pulp MALDI-TOF MS Peptide Profiling
Source: PLoS One. 2011 Feb 25;6(2):e17319. doi: 10.1371/journal.pone.0017319 (PMC3045434; doi:10.1371/journal.pone.0017319)
Supplement: Table S5 — Results of blindly classification of modern mammal individuals by MALDI-TOF MS. (DOC) [file pone.0017319.s007.doc]

|  | **Mammal** | **Species** | **Individuals** | **Teeth** | **Code for blindly classification** | **MALDI Biotyper results** | |
| --- | --- | --- | --- | --- | --- | --- | --- |
|  |  |  |  |  |  | **Species** | **Score** |
| **1** | **Human** | *Homo sapiens* | Woman 1 | F1D1 | N°35 | *Homo sapiens* | 2.518 |
|  |  |  | Man 1 | H1D1 | N°36 | *Homo sapiens* | 2.642 |
|  |  |  | Man 2 | H2D1 | N°37 | *Homo sapiens* | 2.585 |
| **2** | **Rat** | *Rattus rattus* | Rat 36 | R36 | N°21 | Non-classified |  |
|  |  |  | Rat 39 | R39 | N°22 | *Rattus rattus* | 2.059 |
|  |  |  | Rat 40 | R40 | N°23 | *Rattus rattus* | 2.058 |
| **3** | **Guinea-pig** | *Cavia porcellus* | Guinea pig 1 | GP1 | N°17 | Non-classified |  |
|  |  |  | Guinea pig 2 | GP2 | N°18 | *Cavia porcellus* | 2.014 |
|  |  |  | Guinea pig 3 | GP3 | N°19 | *Cavia porcellus* | 2.172 |
|  |  |  | Guinea pig 4 | GP4 | N°20 | Non-classified |  |
| **4** | **Rabbit** | *Oryctolagus cuniculus* | Rabbit 1 | RB1 | N°29 | *Oryctolagus cuniculus* | 2.027 |
|  |  |  | Rabbit 2 | RB2 | N°30 | *Oryctolagus cuniculus* | 2.000 |
|  |  |  | Rabbit 3 | RB3 | N°31 | *Oryctolagus cuniculus* | 2.014 |
| **5** | **Pig** | *Sus scrofa domesticus* | Pig 1 | P1 | N°27 | *Sus scrofa domesticus* | 2.231 |
|  |  |  | Pig 2 | P2 | N°28 | *Sus scrofa domesticus* | 2.278 |
| **6** | **Wild boar** | *Sus scrofa* | Wild boar 1 | WB1 | N°11 | *Sus scrofa* | 2.300 |
|  |  |  | Wild boar 2 | WB2 | N°12 | *Sus scrofa* | 2.266 |
|  |  |  | Wild boar 3 | WB3 | N°13 | *Sus scrofa* | 2.419 |
| **7** | **Cow** | *Bos taurus* | Cow 1 | C1 | N°5 | *Bos taurus* | 2.232 |
|  |  |  | Cow 2 | C2 | N°6 | *Bos taurus* | 2.143 |
| **8** | **Goat** | *Capra hiscus* | Goat 1 | G1 | N°9 | *Capra hiscus* | 2.677 |
|  |  |  | Goat 2 | G2 | N°10 | *Capra hiscus* | 2.540 |
| **9** | **Roe deer** | *Capreolus capreolus* | Roe deer 1 | RD1 | N°32 | *Capreolus capreolus* | 2.573 |
|  |  |  | Roe deer 2 | RD2 | N°33 | *Capreolus capreolus* | 2.057 |
|  |  |  | Roe deer 3 | RD3 | N°34 | *Capreolus capreolus* | 2.485 |
| **10** | **Dromedary** | *Camelus dromedarius* | Dromedary 1 | D1 | N°14 | *Camelus dromedarius* | 2.472 |
|  |  |  | Dromedary 2 | D2 | N°15 | *Camelus dromedarius* | 2.471 |
|  |  |  | Dromedary 3 | D3 | N°16 | *Camelus dromedarius* | 2.479 |
| **11** | **Dog** | *Canis familiaris* | Dog 1 | Dg1 | N°24 | *Canis familiaris* | 2.554 |
|  |  |  | Dog 2 | Dg2 | N°25 | *Canis familiaris* | 2.441 |
|  |  |  | Dog 3 | Dg3 | N°26 | *Canis familiaris* | 2.642 |
| **12** | **Red fox** | *Vulpes vulpes* | Red fox 1 | RF1 | N°1 | *Vulpes vulpes* | 2.189 |
|  |  |  | Red fox 2 | RF2 | N°2 | *Vulpes vulpes* | 2.110 |
|  |  |  | Red fox 3 | RF3 | N°3 | Non-classified |  |
|  |  |  | Red fox 4 | RF4 | N°4 | *Vulpes vulpes* | 1.436 |
| **13** | **Cat** | *Felis catus* | Cat 1 | Ca1 | N°7 | *Felis catus* | 2.074 |
|  |  |  | Cat 2 | Ca2 | N°8 | *Felis catus* | 2.239 |
