# Supplementary material for: Classification of Ancient Mammal Individuals Using Dental Pulp MALDI-TOF MS Peptide Profiling
Source: PLoS One. 2011 Feb 25;6(2):e17319. doi: 10.1371/journal.pone.0017319 (PMC3045434; doi:10.1371/journal.pone.0017319)
Supplement: Table S6 — Results of classification of ancient mammal individuals by MALDI-TOF MS peptide profiling of the dental pulp. (DOC) [file pone.0017319.s008.doc]

| **Site** | **Dating** | **Species** | **Number of Individuals** | **Number of teeth** | **Tooth** | **MALDI Biotyper results** | | | |
| --- | --- | --- | --- | --- | --- | --- | --- | --- | --- |
|  |  |  |  |  |  | **Local modern database reference** | | **Local ancient database reference** | |
|  |  |  |  |  |  | **Species** | **Score** | **Species** | **Score** |
| **Lattes, France** | Middle Age | Pig | 1 | 2 | AP 1 | No reliable classification |  | Ancient sus | 2.315 |
|  |  |  |  |  | AP 2 | No reliable classification |  | Ancient sus | 2.061 |
| **Lattes, France** | Middle Age | Cow | 4 | 4 | AC 1 | *Bos taurus* | 1.624 | Ancient bos | 2.361 |
|  |  |  |  |  | AC 2 | *Bos taurus* | 2.160 | Ancient bos | 2.414 |
|  |  |  |  |  | AC 3 | No reliable classification |  | Ancient bos | 2.530 |
|  |  |  |  |  | AC 4 | No reliable classification |  | Ancient bos | 1.985 |
| **Lattes, France** | Middle Age | Dog | 2 | 4 | AD 1 | *Canis familiaris* | 2.042 | Ancient canis | 2.491 |
|  |  |  |  |  | AD 2 | *Canis familiaris* | 2.321 | Ancient canis | 2.330 |
|  |  |  |  |  | AD 3 | *Canis familiaris* | 2.036 | Ancient canis | 2.487 |
|  |  |  |  |  | AD 4 | *Canis familiaris* | 2.024 | Ancient canis | 2.461 |
| **Lille, France** | XIII_XIV century | Cat | 2 | 2 | AC 1 | No reliable classification |  | Ancient felix | 1.907 |
|  |  |  |  |  | AC 2 | *Felix catus* | 1.850 | Ancient felix | 2.606 |
| **Douai, France** | XVIII century | Human | 2 | 2 | AH 1 | No reliable classification |  | Ancient homo | 2.093 |
|  |  |  |  |  | AH 2 | *Homo sapiens* | 1.325 | Ancient homo | 1.814 |
| **Syria** | 6500 BC | Human | 2 | 2 | AH 3 | No reliable classification |  | Ancient homo | 1.800 |
|  |  |  |  |  | AH 4 | No reliable classification |  | Ancient homo | 2.070 |
| **Rome, Italy, catacombs** | I-III century | Human | 2 | 2 | AH 5 | *Homo sapiens* | 2.362 | Ancient homo | 2.678 |
|  |  |  |  |  | AH 6 | *Homo sapiens* | 1.975 | Ancient homo | 2.582 |
