# Supplementary material for: Classification of Ancient Mammal Individuals Using Dental Pulp MALDI-TOF MS Peptide Profiling
Source: PLoS One. 2011 Feb 25;6(2):e17319. doi: 10.1371/journal.pone.0017319 (PMC3045434; doi:10.1371/journal.pone.0017319)
Supplement: Table S7 — Observed mass to charge ratio of unique species-specific peaks (USSPs) (1000 – 3000 Da) from 13 mammal species. “m/z” values in bold characters have been reported by Buckley M. et al. [7]. (DOC) [file pone.0017319.s009.doc]

| **COW** | | **CAT** | | **GOAT** | |
| --- | --- | --- | --- | --- | --- |
| **m/z** | **Delta m** | **m/z** | **Delta m** | **m/z** | **Delta m** |
| 1088,3531 | 0,36 ± 0,01 | 1565,6834 | 0,47 ± 0,04 | 1779,9525 | 0,54 ± 0,09 |
| 1264,5358 | 0,47 ± 0,02 | 2216,0137 | 0,53 ± 0,03 | 1821,8941 | 0,60 ± 0,06 |
| **1443,6726** | **0,43 ± 0,02** |  |  | 2939,6513 | 0,78 ± 0,31 |
| 1532,7175 | 0,44 ± 0,01 |  |  |  |  |
| 1676,7804 | 0,45 ± 0,02 |  |  |  |  |
| 1783,7755 | 0,52 ± 0,06 |  |  |  |  |
| 1921,9168 | 0,42 ± 0,05 |  |  |  |  |
| 1937,9593 | 0,43 ± 0,03 |  |  |  |  |
| 2253,1452 | 0,47 ± 0,03 |  |  |  |  |
| 2294,108 | 0,46 ± 0,04 |  |  |  |  |
| 2309,0977 | 0,48 ± 0,04 |  |  |  |  |
| 2418,2116 | 0,51 ± 0,04 |  |  |  |  |
| 2565,303 | 0,48 ± 0,05 |  |  |  |  |
| 2644,3584 | 0,49 ± 0,06 |  |  |  |  |
| 2660,3436 | 0,50 ± 0,07 |  |  |  |  |
| 2735,378 | 0,48 ± 0,07 |  |  |  |  |

| **DOG** | | **ROE DEER** | | **GUINEA-PIG** | |
| --- | --- | --- | --- | --- | --- |
| **m/z** | **Delta m** | **m/z** | **Delta m** | **m/z** | **Delta m** |
| 2785,3648 | 0,66 ± 0,06 | 1060,5119 | 0,05 ± 0,04 | 1772,5985 | 0,52 ± 0,04 |
|  |  | 1130,5226 | 0,48 ± 0,03 |  |  |
|  |  | 1309,6568 | 0,58 ± 0,03 |  |  |
|  |  | 1611,7162 | 0,66 ± 0,05 |  |  |
|  |  | 2338,1745 | 0,60 ± 0,07 |  |  |
|  |  | 2438,2798 | 0,61 ± 0,03 |  |  |
|  |  | 2533,3686 | 0,65 ± 0,05 |  |  |
|  |  | 2757,4638 | 0,67 ± 0,03 |  |  |
|  |  | 2983,6772 | 0,60 ± 0,02 |  |  |

| **RAT** | | **RED-FOX** | | **CAMEL** | |
| --- | --- | --- | --- | --- | --- |
| **m/z** | **Delta m** | **m/z** | **Delta m** | **m/z** | **Delat m** |
| 1451,6588 | 0,56 ± 0,04 | 1565,7361 | 0,59 ± 0,08 | 1496,589 | 0,43 ± 0,05 |
| 2014,9626 | 0,57 ± 0,05 | 1655,7488 | 0,53 ± 0,04 | 1634,7595 | 0,44 ± 0,04 |
| 2695,2961 | 0,61 ± 0,06 |  |  | 2006,034 | 0,46 ± 0,04 |
|  |  |  |  | 2043,0622 | 0,72 ± 0,06 |
|  |  |  |  | 2454,3228 | 0,55 ± 0,05 |
|  |  |  |  | 2741,5661 | 0,57 ± 0,06 |

| **WILD BOAR** | | **PIG** | | **RABBIT** | | **HUMAN** | |
| --- | --- | --- | --- | --- | --- | --- | --- |
| **m/z** | **Delat m** | **m/z** | **Delta m** | **m/z** | **Delta m** | **m/z** | **Delta m** |
| 1198,5802 | 0,44 ± 0,06 | 1934,1196 | 0,60 ± 0,05 | 1083,488 | 0,40 ± 0,02 | 1467,7537 | 0,32 ± 0,03 |
| 1422,6603 | 0,44 ± 0,01 | 2027,0281 | 0,57 ± 0,07 | 1191,4568 | 0,53 ± 0,04 | 1533,6058 | 0,34 ± 0,06 |
| 1445,6788 | 0,47 ± 0,06 |  |  | 1277,5677 | 0,40 ± 0,02 | 1623,7253 | 0,31 ± 0,02 |
| 2013,0061 | 0,49 ± 0,06 |  |  | 1291,6095 | 0,40 ± 0,02 | 1742,7811 | 0,32 ± 0,04 |
| 2110,0825 | 0,52 ± 0,07 |  |  | 1353,8018 | 0,59 ± 0,04 | 1812,8048 | 0,30 ± 0,06 |
|  |  |  |  | 1476,6832 | 0,42 ± 0,02 | 1898,9629 | 0,31 ± 0,04 |
|  |  |  |  | 1501,6799 | 0,49 ± 0,02 | 2003,953 | 0,30 ± 0,06 |
|  |  |  |  | 1583,8355 | 0,53 ± 0,04 | 2027,9949 | 0,36 ± 0,09 |
|  |  |  |  | 1860,984 | 0,47 ± 0,05 | 2081,0111 | 0,31 ± 0,06 |
|  |  |  |  | 1947,9491 | 0,43 ± 0,05 | 2090,0478 | 0,35 ± 0,05 |
|  |  |  |  | 2059,0714 | 0,40 ± 0,04 | 2188,0805 | 0,33 ± 0,07 |
|  |  |  |  | **2129,1316** | **0,47 ± 0,04** | 2265,1099 | 0,34 ± 0,09 |
|  |  |  |  | 2530,2685 | 0,51 ± 0,03 | 2281,1185 | 0,42 ± 0,11 |
|  |  |  |  | 2697,4184 | 0,52 ± 0,05 | 2801,4092 | 0,52 ± 0,15 |
|  |  |  |  | 2763,366 | 0,54 ± 0,07 | 2885,5042 | 0,52 ± 0,12 |
|  |  |  |  | 2953,6149 | 0,52 ± 0,05 | **2957,5294** | **0,61 ± 0,16** |
