# Supplementary material for: Classification of Ancient Mammal Individuals Using Dental Pulp MALDI-TOF MS Peptide Profiling
Source: PLoS One. 2011 Feb 25;6(2):e17319. doi: 10.1371/journal.pone.0017319 (PMC3045434; doi:10.1371/journal.pone.0017319)
Supplement: Table S8 — List of the ancient dental pulp specimens used in the study. (DOC) [file pone.0017319.s010.doc]

| Site | **Dating** | **Species** | **Number of teeth** | **Sequence GenBank accession number** |
| --- | --- | --- | --- | --- |
| Lattes, France | Middle Age | Pig | 2 | *Sus scrofa* (95%)  AY237515 |
| Lattes, France | Middle Age | Cow | 4 | *Bos taurus* (95%)  EU807948 |
| Lattes, France | Middle Age | Dog | 4 | *Canis lupus* (95%)  EF689057 |
| Lille, France | XIII_XIV century | Cat | 2 | *Felis catus* (93%)  AB194813 |
| Douai, France | XVIII century | Human | 2 | *Homo sapiens* (93%)  GU903270 |
| Syria | 6500 BC | Human | 2 | - |
| Rome, Italy, catacombs | I-III century | Human | 2 | - |
