# Supplementary material for: Classification of Ancient Mammal Individuals Using Dental Pulp MALDI-TOF MS Peptide Profiling
Source: PLoS One. 2011 Feb 25;6(2):e17319. doi: 10.1371/journal.pone.0017319 (PMC3045434; doi:10.1371/journal.pone.0017319)

Spectrum Analysis Report

Date: 10/18/2010 Time: 10:12

FileName: D:\USERS\Ny\Animaux moderns\MSP\dents humaines\dents humaines\Homme 2\Homme 2 dent 1\0_C6\1\1SRef\pdata\1\1r

Sequence Name:	sp|P02452|CO1A1_HUMAN Collagen alpha-1(I) chain OS=Homo sapiens GN=COL1A1 PE=1 SV=5		
Parentmass:		Mass Error:	
MH+ (mono):	1.008	MH+ (avg):	1.008
Threshold (a.i.):	0.000	Tolerance (Da):	1.000
Number of Peaks:	123	Above Threshold:	
Assigned Peaks:		Not assigned Peaks:	

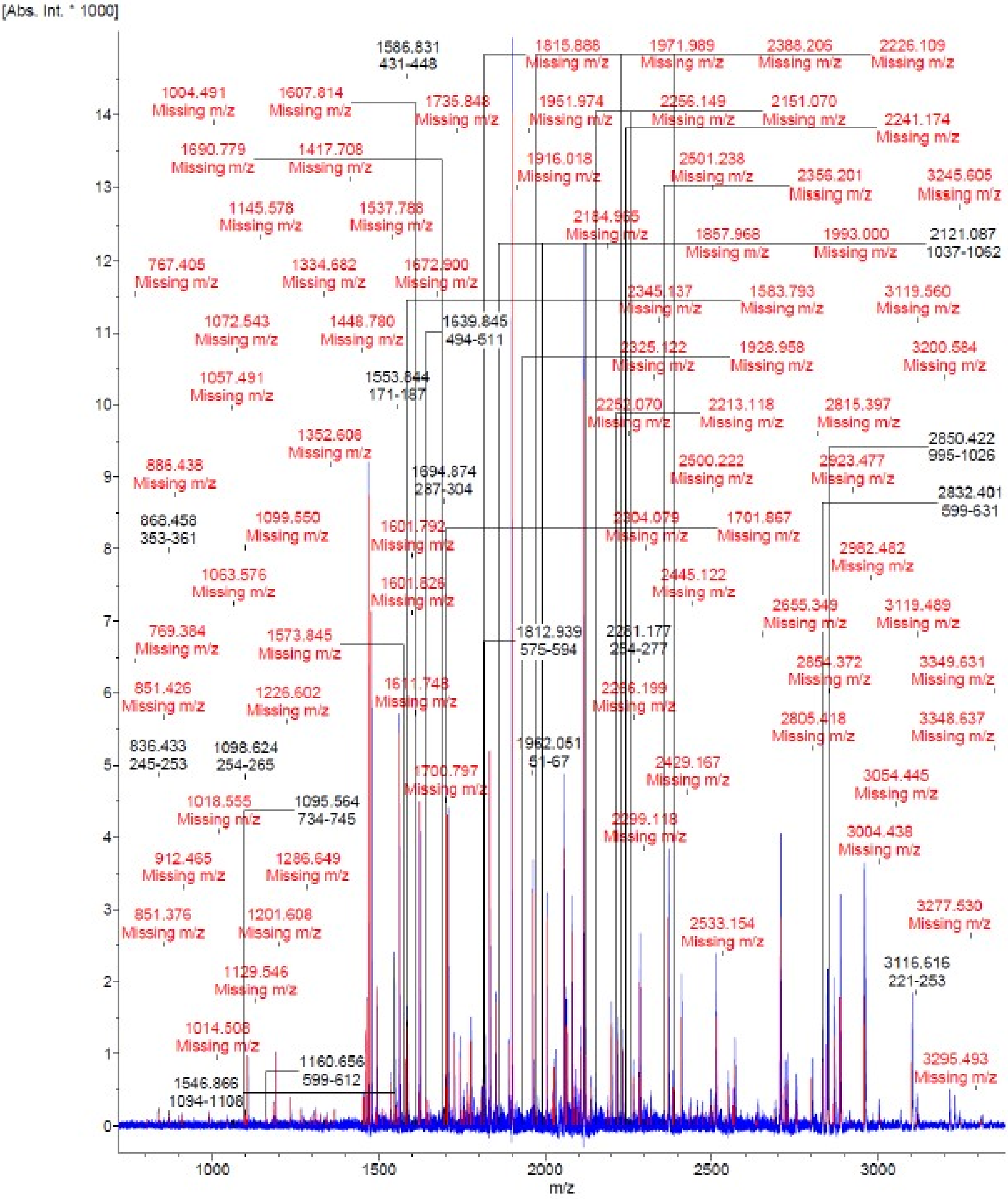

Supplement: Supplementary file 10 [file pone.0017319.s001.doc]
